# Postgraduate education needs of Nurses’ who are caregivers for patients with diabetes

**DOI:** 10.12669/pjms.313.6732

**Published:** 2015

**Authors:** Esra Uğur, Hulya Demir, Elif Akbal

**Affiliations:** 1 Esra Uğur, PhD, Okan University School of Health Sciences, Istanbul, Turkey; 2 Hulya Demir, MSN, Yeditepe University Hospital Nursing Services Directorship, Istanbul, Turkey; 3 Elif Akbal, MSN, Anatolian Health Center Patient Care and Nursing Services Directorship, Kocaeli, Turkey

**Keywords:** Bedside nurse, Diabetes, Education program, Nursing interventions, Patient care

## Abstract

**Objective:**

Diabetic management process requires nurses with expert knowledge and patient care skills. This study was carried out to identify nurses’ diabetic care approaches and their post graduate education needs in order to develop a “Basic Diabetes Patient Care Education Program” in a university hospital in Turkey.

**Methods:**

The descriptive study, using the survey technique, was carried out in a university hospital with 87 bedside nurses who were caring for diabetic patients. Investigators developed data collection tool consisting of closed ended questions and opportunities for open-ended responses.

**Results:**

Among the 87 nurses, 88.5% were staff nurses, and 11.5% were nurse managers. The mean age was 27.41 ± 4.82 and years of professional experience was 6.86 ± 4.23. The 41.4% of nurses stated that they were caring for 1-2 patients with diabetes per week and 72.4% of nurses stated that they had attended an educational session about diabetes after graduation. The 95.4% of nurses reported a need for a continuous education program for diabetes patient care. Medication regimen (69.0%) and special care applications such as wound care (54.0%) were the most needed educational requirements. There were no difference in educational needs based on basic education or years of professional experience (p>0.05).

**Conclusions:**

Nurses caring for patients with diabetes should be supported by orientation, in-service education and continuing education programs. Additionally, the placement of patient care courses for chronic diseases, like diabetes, into the core curriculum of nursing schools would be useful in responding to actual patient care and family needs.

## INTRODUCTION

Diabetes mellitus is a chronic disease that affects 5.6-9.9% of the European population.^1^ It impairs the quality of the life due to complications of vasculopathy and neuropathy in the long term.^2^ Diabetic complications and Type 2 diabetes might be prevented or delayed with life style modification.^3^,^4^ However, patients often have a limited knowledge of a healthy life style and limited insight into their own behavior.^5^ Diabetic patients who did not have diabetic education had a four times increased risk for complications of diabetes.^6^ Acceptance of the disease, insufficient knowledge, and non-compliance were the primary patient related factors that affect the progress of the disease.^7^

At the time of diagnosis complications have not developed. Acceptance of the severity of the disease, as well as adaptation to life style changes needed by the patient and family, might be difficult. Patients require knowledge and motivation to adapt to a new life style. Patients who adapt to a new life style and build changes into their way of life can enjoy their new way of life.^4^ At that point, health care providers have an important role to support and educate the patients about the ways of handling psychosocial problems that arise in livingwithdiabetes.^8^

Nurses are responsible for medication administration, giving care, and educating the patient about the diabetic treatment regimen and life style changes. Expert nursing knowledge and skills are vital to improve outcomes of diabetic care. Especially in the first consultation, nurses have a significant impact on how the patient views the severity of the disease. Giving detailed information about diabetes and its long-term complications, while discussing the importance of medication and life style changes, can build the patient-nurse trust that underlies necessary lifestyle change.

Diabetic patients may be diagnosed while in the hospital for another condition or disease. Thus, all nurses should know the basic approaches to diabetes. However, most nurses do not have the enough background to educate patients about their diabetes, primarily because they have insufficient knowledge or do not trust their knowledge for counseling with diabetic patients.^5^

To increase nurses’ knowledge and expertise about diabetes, various education models can be used. In a chronic disease such as diabetes, interactive and/or simulation education models with case studies may be most useful. Burton and colleagues^1^ suggested an online study program for patient education and health care professional education, with counseling as a part of continuation/specialization studies. These were supportive programs to improve glycemic control and improve quality of life in European partner organizations and Erasmus universities.

In European countries, undergraduate and postgraduate programs of nursing have different educational approaches about caring for patients with diabetes. Education in undergraduate programs for the diabetic patient differs among the countries from 20 hours to 158 hours. Also, while some countries have postgraduate nursing education programs for diabetes management some do not.^1^ In Turkey there are few elective courses specific to diabetic patient care in undergraduate programs. However, most of the hospitals have diabetic patient care education in the orientation program for new nurses. On the other hand, almost every hospital has a diabetic nurse educator who has completed postgraduate courses in diabetic care and follows the role of the diabetic education nurse as described in a Turkish ordinance issued in 2010. There is generally only one diabetic educator in most hospitals with the responsibility of patient education falling to the staff nurse giving care.

Continuing education programs are an important step to keep and improve nurses’ skills and knowledge about diabetes. These programs need to be based on identified nurse needs. Nurses’ self-assessment helps to maintain nursing skills and to explore areas for further development.^9^ The aim of this study was to investigate the nurses’ perceptions on their skills, knowledge and needs in the area of diabetes in a university hospital in Turkey.

## METHODS

This descriptive study was conducted in a university hospital in Istanbul, Turkey. The study received approval from the participating organization’s management. All bedside nurses were considered for participation in the study. Nurses working in the laboratory, operating room or education department were excluded.

Data were collected with a data collection tool, prepared by investigators, between 3-21 January 2011. The sample size was 87 nurses, 88.5% were staff nurse, and 11.5% were nurse managers who also cared for patients. The response rate was 79.81% (n=87) and all participants were informed orally and in written form.

The questionnaire had three major sections. Section 1 included10 demographic questions about the background of the nurses. In section 2, there were 3 questions investigating the diabetic patient care approaches of nurses and their service. In section 3, there were 5 questions examining the educational needs of nurses. The questions were closed with an opportunity for an open-ended response following each section of the questionnaire. The data were coded and analyzed with SPSS 13.0. The number and percentage of the cases, standard deviation, and chi-square were used for the evaluation of the data.

## RESULTS

The mean age of the nurses was 27.41 ± 4.82 and they had 6.86 ± 4.23 years of professional experience. Nearly half, 50.6% (n=44), of them were graduated from high school nursing program. In Turkey high school students may enroll in nursing courses throughout their high school years and subsequently graduate as nurses. Many (41.4%) of nurses reported that they are caring for 1-2 patients with diabetes per week and 72.4% of nurses indicated that they had attended education about diabetes after graduation.

All the nurses followed physician orders for diabetic patients, however, they use a combination of physicians and diabetic nurses for many patients care consultations. A large number 81.6% indicated that for diabetic patient care, they were acting under the direction of the physician’s advice while 77% were combining the advice of physicians with that of the diabetic nurse. The diabetic nurse has specialized knowledge in diabetes, however, in most hospitals because there is only one position for this role, it makes it impossible for the diabetic nurse to meet the demands of the entire hospital. As a result, staff nurses reported that they were primarily planning for education of insulin injections (63.2%) for patients and a regimen of oral anti-diabetes (60.9%), for patients during their discharge period (Table-I). More than 1/3 of the nurses considered themselves to have inadequate knowledge about diabetic ketoacidosis, mechanisms of drug action, types of insulin, HbA_1_C, and special issues (Table-II).

**Table-I:** Nurses’ Diabetes Patient Care Approaches.

	n*	%
*General approaches to the patient care*		
Act in direction of primary doctor	71	81.6
Act in direction of diabetes nurse’s suggestions	67	77.0
Continue the routines of patient before hospitalization	4	4.6
Don’t give special care if the main reason is not DM	2	2.3
*Discharge education planning*		
Oral anti diabetics	53	60.9
Insulin injection	55	63.2
Glucometres use and blood sugar measurement	51	58.6
Foot care	41	47.1
Management of diabetic complications	44	50.6
Routine doctor controls	52	59.8

* More than one choice was selected.

**Table-II:** Issues with which nurses feel uncomfortable while caring for the diabetes patient.

Issues	n*	%
*Answering the questions of patients and relatives*		
Planning drug times	22	25.3
Insulin injection method and equipment	16	18.4
Special care applications	20	23.0
Routine doctor controls	12	13.8
Daily life activities	10	11.5
Management of complications	21	24.1
Different treatment choices	25	28.7
Special issues (pregnancy, preop period etc.)	34	39.1
*Explaining the blood test results*		
Blood glucose test	8	9.2
HbA1C	30	34.5
*Management of diabetic emergencies*		
Hypoglisemia	8	9.2
Diabetic ketoacidosis	38	43.7
*Oral anti diabetic drug applications*		
Planning drug hours (before and after meals)	19	21.8
The mechanism of drug action	32	36.8
Duration of drug action	28	32.2
*Insulin applications*		
Types of insulin	34	39.1
Injection techniques	5	5.7
Planning application hours	17	19.5
Insulin infusions	18	20.7
*Special care applications*		
Foot care	10	11.5
Wound care	24	27.6
Skin care	9	10.3
Oral and dental health	9	10.3

* More than one choice was selected.

A high percentage of nurses (95.4%)wanted the focus for a continuing education program to be on the topics of medication regimen (69.0%), special care applications such as wound care (54.0%) and nursing care in general (52.9%) (Table-III). There was no statistical significance in the findings on educational needs and feelings of discomfort during the care of diabetic patients (p>0.05) when comparing nurses based on graduate degree and/or length of time of professional experience.

**Table-III:** Subjects of an education program that nurses need.

Subjects	n*	%
Physiopathology of DM	23	26.4
Diagnosis and treatment approaches	30	34.5
Nursing care in DM	46	52.9
OAD and insulin use	60	69.0
Special care applications (ex.woundcare)	47	54.0
Patient education	13	14.9

* More than one choice was selected.

## DISCUSSION

This study indicates the importance of continuous education program for nurses to improve healthcare outcomes. In our study, almost 2/3 of the subjects were teaching insulin injection in the discharge period, and 39% of them have reported inadequate knowledge on the types of insulin. This study also reveals inadequate knowledge and skills in general diabetic nursing care, the use of anti-diabetics and special care such as wound care and nutrition. These findings argue for complete and comprehensive diabetic education for bedside nurses.

Nurses, as primary health care providers, have important roles in the patient’s adaptation and acceptance of life style modification in chronic diseases. Nurses need to incorporate skills of leadership, communication, counseling, teaching and research into their practice in order to improve patient care.^3^ In the primary care units, screening for diabetes and providing illness prevention are performed by the nurses.^10^ In general most of the diabetic patients receive support in the primary care units from the nurses who have graduated from the nursing school, but do not have any postgraduate education about diabetes. They have the basic knowledge about diabetes and its complications, but their knowledge of diabetic patient care is insufficient.^10^

Bedside nurses have the greatest access to the patient and their approach to the patient on diabetes influences the success of diabetic patient care.^9^ Consequently, all bedside nurses need to have adequate diabetic patient care knowledge including education on diet and insulin therapy management.^11^ To improve outcomes of the diabetic patient care, it is critical that, all nurses should be participated in continuing diabetic education programs.

Tschannen and colleagues^12^ reported that 81% of the nurses could describe the pathophysiology of diabetes, 90% could describe the treatment, and 81% could describe how to use the patient education materials. However 48% of the nurses and nursing students could not define the correct treatment of hypoglycemia.^11^ The gap between the schools and work-life is one of the reasons of the feeling insufficient knowledge by the health-care providers. Continuous education programs are important step to transfer the basic knowledge to routine practice. Computer based education methods are also an effective method for educating nurses, one which works in different departments and on various shifts, with a base in evidence based practice.^13^ Online education has the advantage of access to information at flexible times and had a similar knowledge score when compared with the didactic session.^14^

Graduating from a nursing program does not necessarily mean that they have sufficient knowledge about diet, knowledge that includes the carbohydrate content of foods and the relationship of food intake to insulin needs. Mensing and colleagues^15^ suggest that dietary education should be given by the diabetes educators or registered dietitians. Some nurses agree that dietary education is the dietician’s task.^5^ In contrast, Carney and colleagues^11^ reported that 70% of nurses and nursing students believe that nurses have an important role in patient nutritional education. Placing dietary education within the nurses’ continuing education programs could improve competence, self-confidence, trust between the patient and nurse, and improve patient outcomes.

Overall, diabetic self-management education is the cornerstone of the outcomes of diabetic patients.^15^ Education of patients with diabetes and their families allows them to become active in the management of this disease.^7^ The study reported by El-Dairawi et al.^16^ notes that the education level of the nurses was positively correlated with their knowledge about diabetes. In the study of McDonald and colleagues^7^ 84% of 136 nurses reported the need for more education to improve diabetic patient care. Jansink et al.^5^ reported that in the life style counseling process, nurses do not have the time or skills for teaching or counseling patients, and nurses need to improve their counseling skills in order to impact patient motivation.^5^ Professional staff that participates in continuing diabetes education and behavioral interventions can improve their skills, attitudes and outcomes of diabetic care.^15^ Supportive role of the healthcare providers and follow-up visits might be helpful to improve patients factors.

Problem-based learning and simulation are also helpful to develop necessary skills in diabetes management and the management of other chronic diseases.^12^ Strang et al.^17^ suggested Diabetes Conversation Maps. Conversation Maps are a visual way to stimulate and focus discussion on living with diabetes. The maps are not a game, but are a way for participants to discover facts about diabetes for themselves.

It is possible to establish a career program in diabetic nursing, one that will meet the expectations of patients and improve the outcomes of patients with diabetes. Diabetes Management Mentor course improves outcomes of diabetic patient care with increasing bedside nurses’ knowledge and skills in diabetes management.^18^ Diabetes Management Mentors are empowered with the skills of teaching, inspiring and nurturing other bedside nurses through monthly continuing educational programs.^18^

El Dairawi et al.^16^ reported that in the hospitals, that employ diabetes educators, hospital nurses are not actively involved in teaching the diabetic patient and do not attempt to increase their own level of diabetic patient care. It is necessary to clarify the responsibilities of nurses for diabetic patient care in each part of healthcare organizations.^19^ In most hospitals motivation and training of the bedside nurses is important to improve diabetic patient care. In the study reported here, the University hospital has one diabetic nurse educator and she is responsible for patient education. However, bedside nurses are responsible for diabetic patient care and impart education. Nurses’ perceptions are critical to improving their ability to offer competent diabetic patient care. In this study the authors have described their findings and recommend a strong continuing education program in diabetic education for all bedside nurses.

This study was conducted in a university hospital. Nurses joined the study voluntarily. The data was collected by the self-reported questionnaire. Since the questionnaire included the names and the academic degree it is possible that there is a response bias. The study results cannot be generalized to all nurses, or even to all Turkish nurses, however the study helps to identify the gaps in the routine practice of diabetic patient care.

## CONCLUSION

The findings of this study suggest that the most frequent issues nurses experience in diabetes management are discomfort in the areas of medication and diabetic emergency management. Placing courses related to diabetes and other chronically ill diseases into core curriculum of nursing schools will be useful to improve patient outcomes. In addition, post-graduate education programs including orientation, in-service education and continuing education programs should support nurses caring for diabetic patients.
